# A data-driven computational methodology for assessing ventricular ablation procedures

**DOI:** 10.1007/s10237-026-02073-7

**Published:** 2026-06-03

**Authors:** Filippo Caruso Lombardi, Anna Crispino, Bich Lien Nguyen, Nicola Galea, Alessandro Loppini, Simonetta Filippi, Francesco Viola, Alessio Gizzi

**Affiliations:** 1 Gran Sasso Science Institute (GSSI), Viale Luigi Rendina 26, 67100 L’Aquila, Italy; 2 Department of Engineering, Università Campus Bio-Medico di Roma, Via A. del Portillo 21, 00128 Rome, Italy; 3 Department of Cardiology, Sapienza University of Rome, Viale del Policlinico 155, 00161 Rome, Italy; 4 Department of Radiological, Oncological and Pathological Sciences, Sapienza University of Rome, Viale Regina Elena 324, 00161 Rome, Italy; 5 Department of Medicine and Surgery, Università Campus Bio-Medico di Roma, Via A. del Portillo 21, 00128 Rome, Italy; 6 INFN-Laboratori Nazionali del Gran Sasso, Assergi, Italy

**Keywords:** Ventricular tachycardia, Electroanatomical mapping, Cardiac ablation, Patient-specific modeling, Computational electrophysiology, GPU acceleration

## Abstract

Ventricular tachycardia following myocardial infarction is often sustained by complex reentrant circuits that are challenging to characterize and treat using conventional electroanatomical mapping. Computational modeling provides a powerful complementary approach to understanding conduction pathway dynamics more effectively and supporting ablation strategies. Here, we present a reproducible and data-driven clinically guided computational framework for the retrospective analysis of post-infarction ventricular tachycardia and ablation procedures. The method integrates patient-specific electroanatomical mapping data—including local activation times, voltage maps, and electrograms—to build a personalized model that captures both structural and functional remodeling via a viability-based scalar field. A novel calibration procedure is introduced to locally estimate tissue conductivity, enabling accurate reproduction of observed activation patterns. The model is used to simulate arrhythmia inducibility and sustainability, and to retrospectively evaluate the impact of clinical radiofrequency ablation, accounting for lesion size and transmurality. In silico exploration of alternative ablation strategies is also performed to minimize lesion volume while maintaining arrhythmia suppression. The entire workflow is designed for rapid execution using a GPU-accelerated monodomain solver and is fully compatible with existing clinical practices, offering a practical tool for substrate interpretation and patient-specific ablation planning.

## Introduction

**Fig. 1:**
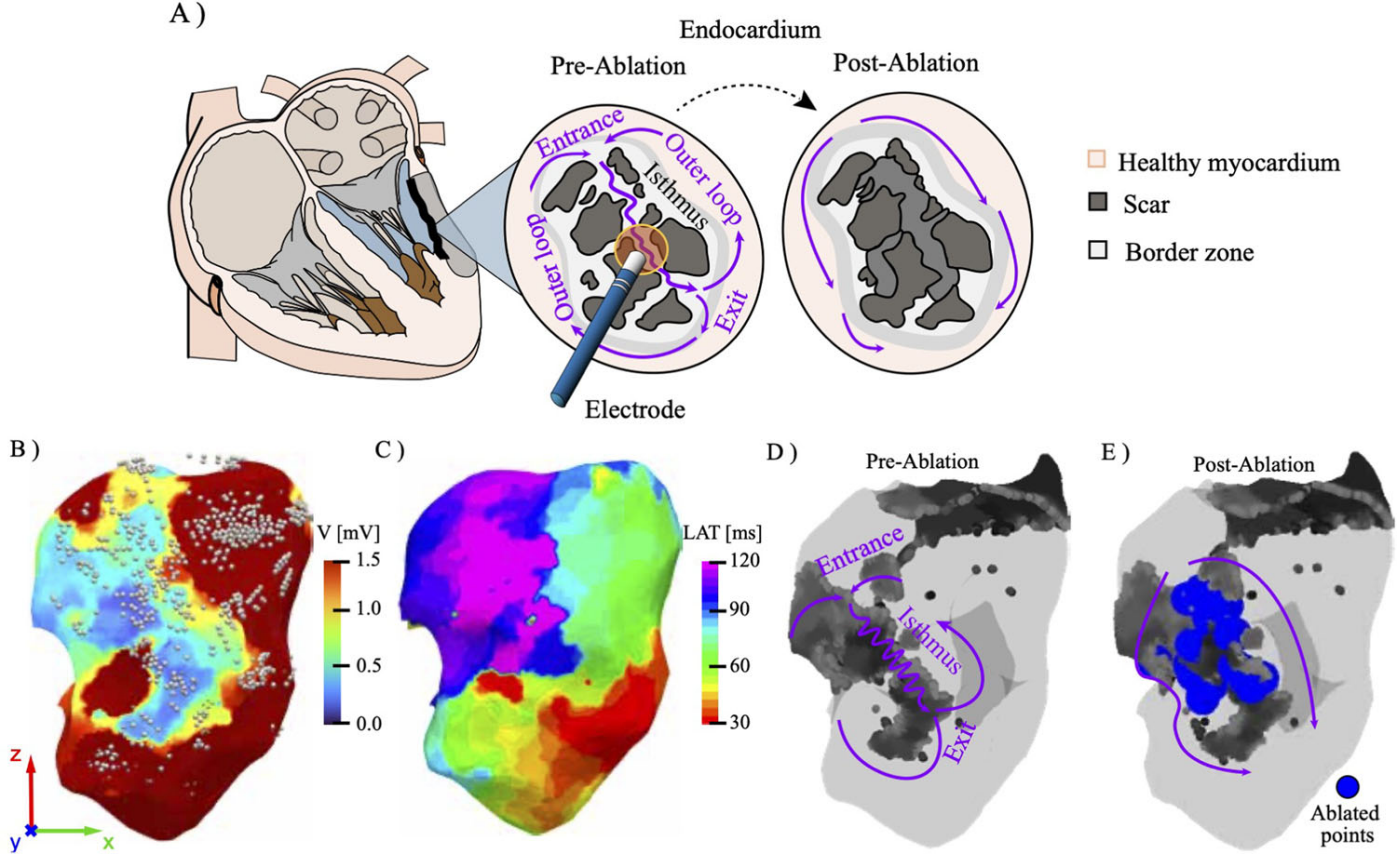
**A** Schematic of ventricular endocardial surface in a post-infarction patient, illustrating the reentrant VT circuit (pre-ablation) case and its suppression (post-ablation). Isthmus is bounded by conduction block or slowed conduction zones: healthy myocardium (pink), border zone (gray), and dense scar (black). Purple arrows indicate the propagation loop involving entrance, isthmus, and exit sites. **B** Corresponding CARTO sinus rhythm maps displaying bipolar voltage, with overlaid positions of the mapping electrode (white dots) during data acquisition. **C** Local activation time (LAT), where low-voltage regions ($\le 1\,\text {mV}$) and delayed activation times overlap with anatomical isthmus. **D** CARTO-based reconstructions showing electrical isolation of the reentrant pathway, with **E** ablated regions highlighted in blue

Cardiac ventricular tachycardia (VT) is a life-threatening arrhythmia, frequently associated with ischemic cardiomyopathy and prior myocardial infarction (MI) (Kahle et al. 2021; Nguyen et al. 2015; Tung et al. 2020). The underlying mechanism in post-infarction VT is typically macroreentry, where the reentrant circuit propagates through narrow isthmuses of surviving myocardium embedded within heterogeneous fibrotic scar (Fig. 1A) (Ciaccio et al. 2001, 2005, 2007). The identification of the critical isthmus in patients with chronic ischemic heart disease remains challenging, primarily due to unstable clinical conditions. Despite the efficacy of implantable cardioverter-defibrillators in reducing mortality (Devore and Sm 2019; Hohnloser et al. 2004), VT recurrence remains frequent and profoundly impacts patient quality of life due to recurrent shocks and re-hospitalizations (Guandalini et al. 2019).

Catheter-based radiofrequency ablation (RFA) is the gold-standard therapy in this setting, typically guided by electroanatomical mapping (EAM) (Miller and Zipes 2002; Tung et al. 2010) (Fig. 1A). Conventional EAM integrates bipolar voltage amplitude (Fig. 1B) and local activation time (LAT) (Fig. 1C) to visualize conduction abnormalities on a three-dimensional cardiac geometry (Sanjiv and Roy 2024). Low-voltage regions are indicative of scarred myocardium, while channels of intermediate or late potentials suggest proarrhythmic conduction isthmuses. On EAM maps, isthmuses typically appear as narrow corridors of delayed activations surrounded by dense scar, which are the primary targets for ablation (Calkins et al. 2000). However, the mapping of VT circuits can be hindered by non-inducibility, hemodynamic instability, or polymorphic morphologies (Aliot et al. 2009; Marchlinski et al. 2000; Uzelac et al. 2022). Moreover, limitations in catheter contact or mapping resolution can obscure mid-myocardial or epicardial substrates, leading to incomplete ablation and VT recurrence (Guandalini et al. 2019; Stevenson and Soejima 2007).

To overcome these limitations, substrate-based ablation strategies have gained prominence. These techniques target abnormal electrograms during sinus or paced rhythm, such as late potentials, local abnormal ventricular activities, and regions showing slow conduction or fractionated electrograms (Aronis et al. 2020; Guandalini et al. 2019; Irie et al. 2015). Substrate mapping allows identification of isthmus sites, even in the absence of inducible VT, and is particularly valuable in cases with multiple or unstable arrhythmias. Importantly, substrate mapping is often complemented by pace mapping for localization of VT exit and isthmus sites. Pace mapping during sinus rhythm plays a crucial role in delineating reentry circuit isthmuses. When a pacing site is located near the exit of an isthmus, the resulting QRS morphology may closely replicate that of the clinical VT. Furthermore, the stimulus-to-QRS interval provides insight into conduction delay, with longer intervals suggesting proximal pacing within a slowly conducting isthmus channel (Brunckhorst et al. 2004). This approach, when integrated with 3D electroanatomical maps, facilitates a semi-automated identification of critical channels for ablation (Fig. 1A).

In parallel, advances in computational modeling provide powerful complementary tools for simulating arrhythmic propagation and improving VT circuit visualization (Niederer et al. 2019; Plank et al. 2008; Cluitmans et al. 2024). Patient-specific models constructed from imaging data and EAM-derived voltage maps can simulate electrophysiological activation under various pacing protocols, enabling prediction of reentry circuits and ablation outcomes (Herrera et al. 2022; Jaffery et al. 2024). These models account for tissue heterogeneity, anisotropic conduction, and scar distribution—critical features for accurately reproducing clinical arrhythmias. A variety of energy delivery technologies and strategies are also being explored to improve lesion depth and transmurality, especially for intramural substrates inaccessible to standard endocardial ablation. These include simultaneous bipolar ablation, needle-tipped catheters for intra-myocardial radiofrequency delivery (Bianconi et al. 2025; Petras et al. 2022). While promising, these techniques still require computational optimization and validation for safety and efficacy before routine clinical adoption.

In particular, the development and calibration of such models remain inherently challenging (Pagani et al. 2021; Trayanova et al. 2024) as the electrophysiological properties can fluctuate on a beat-to-beat basis, complicating the selection of representative clinical input data. Moreover, model validation is hindered by the limited availability of comprehensive experimental datasets, which restricts direct assessment of predictive accuracy. In this context, the present work contributes to ongoing efforts aimed at defining robust criteria for predicting individual patient response to ablation therapy.

Within this framework, we propose a reproducible strategy for tuning patient-specific electrophysiological models by leveraging all available CARTO-derived electroanatomical data—including EGMs, LAT maps, and voltage-based substrate reconstructions. Rather than pursuing anatomically exhaustive reconstructions from MRI (Boyle et al. 2019; Gillette et al. 2022; Vadakkumpadan et al. 2014), we emphasize interpretability and slow conduction patterns reconstruction, aiming at the integration into clinical workflows. Particular attention is given to the identification of conductivity and excitability patterns in the critical zones, where arrhythmia is most likely sustained. The model tuning is performed by matching simulated LAT maps and electrograms to clinical observations, allowing for physiologically consistent reproduction of activation dynamics.

In the following, we present a data-driven voltage-guided, quasi-automated framework that enables retrospective assessment of ablation strategies and isthmus targeting, based on data acquired during routine ablation procedures. By inducing arrhythmias through a standardized S1–S2 stimulation sequence, in Sect. 3.1 we identify critical conduction pathways and their role in sustaining scroll waves. In Sect. 3.2, the ablation procedure applied to the patient (and recorded in the CARTO clinical data) is reproduced numerically, thus analyzing its capability in suppressing arrhythmias depending on the lesion size. Driven by these results, in Sect. 3.3 novel ablation strategies reducing the total ablation volume are investigated in silico foreseeing clinical optimization. The proposed methodology is implemented within an in-house bidomain/monodomain solver which is GPU accelerated and that can solve the ventricular electrophysiology within few minutes (Del Corso et al. 2022), thus allowing for fast calibration of the model and rapid testing of different ablation strategies.

## Methods

### Case of study

We retrospectively analyze the case of a male patient with ischemic cardiomyopathy and recurrent VT, admitted for advanced arrhythmia management following multiple hospitalizations for heart failure. The patient had a history of multi-vessel coronary artery disease, previously treated with percutaneous coronary interventions and drug-eluting stents. Cardiac magnetic resonance imaging revealed transmural infarction of the infero-lateral and apical segments, with extensive late gadolinium enhancement in the infero-posterior-lateral wall, consistent with chronic infarction and myocardial remodeling.

Electrophysiological mapping and VT ablation after a written informed consent was obtained. A 3D electroanatomical map (CARTO 3 system) of the endocardial left ventricle wall was acquired in sinus rhythm under right ventricle pacing protocol. Substrate and activation maps were collected. Ultra-high density electroanatomical reconstruction of the left ventricle was conducted, using more than 1000 mapping points. Low-voltage areas suggest endocardial–myocardial scar, defined as the endocardial bipolar voltage $\le 1$ mV. Voltage mapping demonstrated a large infero-lateral scar with a heterogeneous border zone, including areas of delayed and fractionated potentials.

Radiofrequency ablation was performed using an irrigated catheter (35–50$^\circ$C, 30–40 W), targeting slow conduction channels identified during sinus rhythm. Post-ablation mapping confirmed substrate homogenization and non-inducibility of VT.

### Patient-specific computational model

**Fig. 2:**
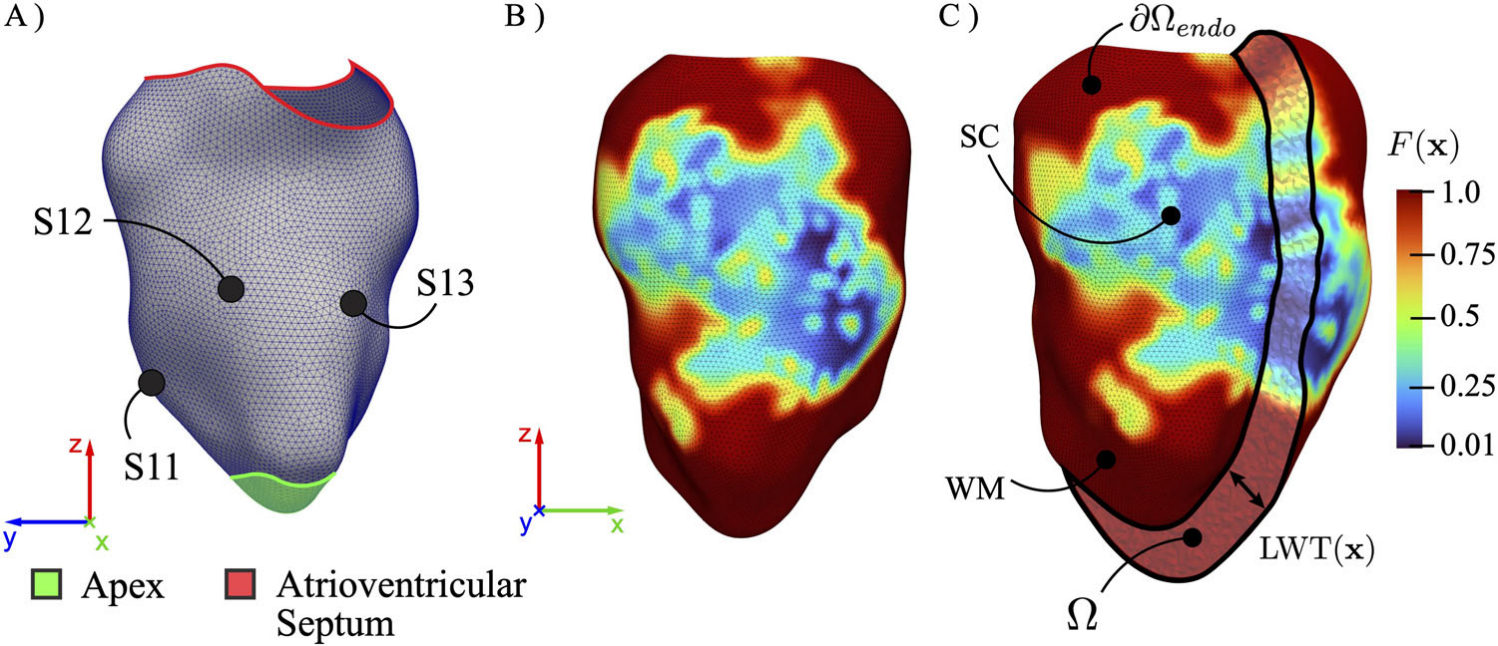
Schematic overview of the pipeline used to obtain the ventricular computational domain. **A** Regularized left ventricular geometry: Black dots indicate the locations of the three S1 stimuli ($\text {S1}j$, $j \in \{1, 2, 3\}$) used to reproduce sinus endocardial activation. **B** Impairing function $F(\textbf{x})$ distinguishing the scar core (dark blue) from the working myocardium (red). **C** Detail of the local wall thickness LWT$(\textbf{x})$ with respect to $F(\textbf{x})$

We propose a semi-automated pipeline for generating a patient-specific electrophysiological model of the infarcted left ventricle (LV) (see Fig. 2). The pipeline is here applied to a patient-specific dataset containing electroanatomical data of MI, according to the CARTO 3 mapping system.

A critical step of the procedure is the identification of the infarcted myocardial tissue within the LV anatomy. This is achieved by analyzing the peak-to-peak amplitude of intracardiac electrograms (EGMs), which serves as a reliable indicator for distinguishing electrically inert scar regions from viable myocardium. The resulting voltage map $\text {V}(\textbf{x})$ (see Fig. 1B) is generated using the OpenEP MATLAB suite (Williams et al. 2021), which interpolates the discrete EGM amplitudes from the registration points (reported as white dots in Fig. 1B)—onto the left ventricular endocardial surface. The voltage map shows a low-voltage region, corresponding to the fibrotic core of MI. Moreover, the CARTO system provides a map of local activation time, see Fig. 1C, where the region $\text {LAT}(\textbf{x})>125\,\text {ms}$ is well correlated to the morphology of the scar that is observed in the voltage map.

To ensure a computational domain that is sufficiently regular for numerical simulations, a Laplacian smoothing filter is applied to the raw patient-specific geometry contained in the CARTO dataset (Fig. 1B). This process yields a regularized representation of the endocardial surface (Fig. 2A), free from spurious geometric discontinuities and small-scale curvatures that could introduce numerical artifacts.

The presence of scar tissue alters the local electrophysiological properties of the myocardium. To incorporate such tissue heterogeneity into the computational model, we introduce a spatially varying impairing function $F(\textbf{x})$ defined on the endocardial surface. This field is directly informed by clinically acquired EGM voltage measurements, such that regions with reduced bipolar EGM amplitude are interpreted as electrically remodeled tissue. This scalar field is defined through the peak-to-peak amplitude of intracardiac bipolar EGMs as $F(\textbf{x}) = (\min (\text {V}(\textbf{x}),\text {V}_{th})/\text {V}_{th})^2$, where $\text {V}_{th} = 1~\text {mV}$ is a threshold value adopted to distinguish between regions of low-amplitude associated with dense fibrotic scar tissue from the electrically active myocardium. Specifically, $F(\textbf{x}) = 1$ in working myocardium (WM), whereas $F(\textbf{x}) \ll 1$ within the scar core (SC). This formulation enables the spatial heterogeneity of the myocardial substrate to be directly encoded from clinically acquired voltage data, through the resulting field $F(\textbf{x})$ reported in Fig. 2B.

#### Altered Myocardial Thickness

A well-known challenge in building a patient-specific numerical model of the infarcted myocardium is that clinical imaging provides only endocardial surface data without accounting for structural remodeling, i.e., altered local myocardial wall thickness (LWT) (Sacristan et al. 2025). In order to distinguish between the infarcted region (thinner wall) and the working myocardium (thicker wall), the impairing function $F(\textbf{x})$ is used to define a spatially varying $\text {LWT}(\textbf{x})$ as:

1 $$\begin{aligned} \text {LWT}(\textbf{x}) = \gamma _0 \left( 1 + \delta _t F(\textbf{x}) \right) \end{aligned}$$

where $\gamma _0$ represents the baseline transmural thickness of the myocardium (Rodrigues et al. 2021), and $\delta _t \simeq 0.25$ is selected to ensure that $\text {LWT}(\textbf{x}) \approx 10\ \text {mm}$ in WM, corresponding to $F(\textbf{x}) = 1$. The resulting distribution of $\text {LWT}(\textbf{x})$ varies smoothly across the LV, transitioning from WM through the SC, see Fig. 2C. This approach provides a three-dimensional representation of the structurally remodeled ventricle, hereafter denoted as $\Omega$, which corresponds to our computational domain. The scalar field $F(\textbf{x})$ is then extended to $\Omega$ by assigning to any myocardial cell (within $\text {LWT}(\textbf{x})$) the corresponding endocardial value.

The maximum myocardial thickness in WM is set to $10\text {mm}$ as it aligns with average MRI data of the patient. In the following, we focus on this configuration to better analyze the effect of the ablation radius. Notably, we deliberately avoid reconstructing the full ventricular geometry from MRI data in order to streamline the workflow, reduce computational costs, and enable faster simulations—while still achieving comparable outcomes. Available MRI data are only used for retrospective validation, see Appendix A.

### Electrophysiology model incorporating the scar

The cardiac electrophysiology is solved via the monodomain model:

2a $$\begin{aligned} &  \frac{\partial U_m}{\partial t} = \nabla \cdot \left[ \textbf{D}(F(\textbf{x})) \nabla U_m \right] + I_{ion}\left( U_m,\mathbf {\phi }\right) +I_s(\textbf{x},t),\nonumber \\ \end{aligned}$$

2b $$\begin{aligned} &  \frac{\textrm{d} \mathbf {\phi }}{\textrm{d} t} = \textbf{G}\left( U_m, \mathbf {\phi },F(\textbf{x})\right) , \quad \text {in} \quad \Omega , \end{aligned}$$

where $U_m$ is the transmembrane potential in $[\text {mV}]$ and $I_{ion}$ in $[\text {mA/mm}^{2}]$ is the total ionic current given by the cell model $\textbf{G}$, having state vector $\phi$. $\textbf{D}(\textbf{x}) \equiv \textbf{D}(F(\textbf{x}))$ is the heterogeneous tissue diffusivity tensor in $\text {[mm}^{2}/\text {ms]}$ and $I_s$ is a prescribed input current introduced to initiate electrical propagation. The forcing term $I_s$ in Eq. (2a) consists of a periodic electrical stimulus, hereafter denoted as S1. To emulate physiological ventricular activation and compensate for the absence of an explicit Purkinje network, the S1 stimulus is subdivided into three spatially distinct sub-stimulations (S11, S12, and S13, Fig. 2A), collectively designed to approximate the clinically recorded endocardial conduction patterns. Specifically, the activation originates in the superior portion of the interventricular septum, propagates toward the ventricular apex, and then ascends toward the basal regions. The system is completed by insulating conditions ($\nabla U_m \cdot \textbf{n}=0$) on the endocardial and epicardial surfaces, as well as on the atrioventricular fibrous skeleton (highlighted in red in Fig. 2A).

Importantly, the presence of the fibrotic scar is incorporated in the monodomain equations through the impairing function $F(\textbf{x})$, which perturbs the cell model in the less-viable tissue (as detailed in Sect. 2.3.1) and it modulates the tissue diffusivity tensor $\textbf{D}(F(\textbf{x}))$ to capture local alterations of the conduction velocity (see Sect. 2.3.2)

The system (2) is solved numerically using an in-house Fortran code, which is well-suited for solving reaction–diffusion dynamics in anatomically realistic domains (Del Corso et al. 2022; Viola et al. 2023). The solver is based on a cell-centered finite volume method, which has the main advantage of having a diagonal mass matrix and when combined with an explicit temporal scheme, it allows to march the equations in time without solving any linear system (differently from standard finite-element methods which call for the solution of large linear systems at any time step). Furthermore, the solver has been GPU-accelerated and the ventricular electrophysiology can be solved within few minutes. The computational domain $\Omega$ is discretized using a tetrahedral mesh with an average cell size of $h= 0.22 \text { mm}$ and temporal integration is performed using a constant time step of $\Delta t = 0.01 \text { ms}$. The wall clock time to solve $100\,\text {ms}$ of the cardiac activity on a single GPU device (A100 by Nvidia) is $54.1\,\text {s}$.

#### Altered cell model

The cell model $\textbf{G}(\textbf{x}) \equiv \textbf{G}(F(\textbf{x}))$ in Eq. (2b) governs the transient ionic fluxes and is coupled to the monodomain equation  (2a) through the ionic current $I_\textrm{ion}$. We adopt the four-variable minimal model developed by Bueno-Orovio et al. (Bueno-Orovio et al. 2008), which offers a well-balanced trade-off between biophysical fidelity and computational efficiency.

The model is initialized using the parameter set described in Fenton et al. (2013), which characterizes endocardial ventricular behavior under physiological conditions ($37^\circ \text {C}$). To incorporate infarct-induced electrophysiological remodeling, we locally modify a subset of model parameters within the MI through the impairing function $F(\textbf{x})$. Hence, the model parameters reported in Table 1 vary smoothly over the myocardium $\Omega$ as $p=p^*\left[ 1+\delta _p (1-F(\textbf{x})) \right]$ where $p^*$ is the reference parameter value and $\delta _p$ governs the perturbation amplitude. The selected parameter subset is chosen to reproduce key electrophysiological features of infarcted tissue, such as action potential duration (APD) prolongation and a reduced action potential overshoot (Varró et al. 2021). The resulting perturbed parameters are listed in Table 1, while the full electrophysiological model formulation is reported in Appendix B.

**Table 1:** Perturbed parameters and corresponding perturbation amplitudes of the four-state minimal model

Parameter	$\theta _v$	$\theta _w$	${\tau _{w1}}^-$	${\tau _{w2}}^-$	$\tau _{fi}$	$\tau _{so1}$	$\tau _{so2}$
$p^*$	0.3	0.13	40	115	0.10	40	1.2
$\delta _p$	0.08	0.16	0.16	0.16	0.16	0.16	0.16

**Fig. 3:**
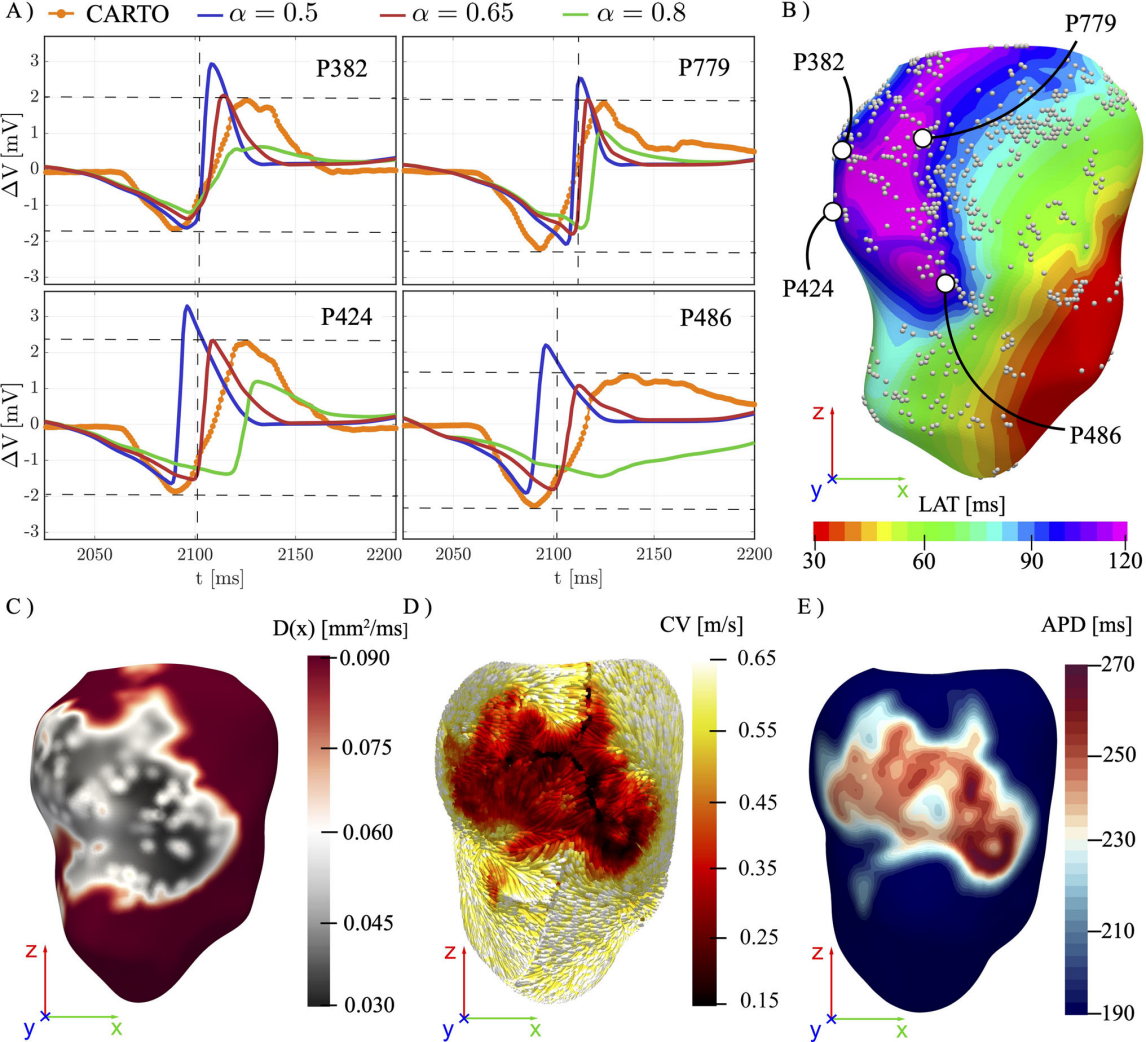
**A** Comparison between in vivo and in silico EGMs (for $\alpha =0.5, 0.65, 0.8$) in four representative measurement points around the scar region. Horizontal and vertical dashed lines indicate, respectively, the maximum/minimum amplitude and deflection points of the EGM. **B** In silico LAT map from the monodomain simulations, with superimposed measurement locations of the EGMs taken from CARTO. The larger circles indicate the four locations corresponding to the EGMs reported in (**A**). **C** Electrical diffusivity field $\textbf{D}(\textbf{x})$ where the gray region corresponds to the scar core. **D** CV streamlines colored by its magnitude. **E** APD map on the ventricle endocardium

#### Altered electrical diffusivity

Cardiac myocytes are organized in fiber-like microstructures leading to a faster conduction along the fiber direction compared to their transverse section, namely the sheet and sheet-normal directions, which correspond to the principal directions of the tensor $\textbf{D}(\textbf{x})$. Moreover, the myocardial fiber arrangement following MI strongly depends on the morphology of the scar (Nielles-Vallespin et al. 2020; Winklhofer et al. 2014; Wu et al. 2006), with a progressive disarrangement leading to slower conduction velocities approaching the SC. Owing to the lack of such data for the patient under study and highlighting the critical role of dispersion of repolarization in pathological hearts (Loppini et al. 2019; Watanabe et al. 2001), the left ventricular myocardium is modeled as an isotropic, spatially heterogeneous conductive medium, where electrical diffusivity decreases linearly via the impairing function $F(\textbf{x})$ as:

3 $$\begin{aligned} \textbf{D}(\textbf{x}) = D(F(\textbf{x})) \textbf{I} =\beta \left[ 1 - \alpha (1 - F(\textbf{x})) \right] \textbf{I} \quad \text {in } \, \Omega , \end{aligned}$$

where $\beta =0.09\,\text {mm}^2/\text {ms}$ is set to yield a $\text {CV}\approx 0.6~\text {m/s}$ in WM, and $\alpha$ is a weight parameter reducing the diffusivity within the scar tissue via $F(\textbf{x})$; $\textbf{I}$ is the identity tensor. The proposed modeling approach ensures that the diffusivity tensor is spatially distributed, thereby reproducing the conduction delay or block typically associated with fibrotic substrates.

Patient data recorded under sinus rhythm (at 60 bpm) are used to calibrate the remaining free parameter $\alpha$ in Eq. (3). The procedure compares in vivo EGMs with those obtained in silico, following the approach proposed in Cabrera-Lozoya et al. (2017). The white dots in Fig. 1B represent the available locations of EGM, while a subset of in silico EGMs around the scar is reported in Fig. 3A, for $\alpha =0.5,\,0.65,\,0.8$ and compared with their in vivo counterpart. Increasing $\alpha$ results in (i) an attenuated EGM amplitude and (ii) progressively delayed main deflections, owing to slower local conduction. This effect follows a monotonic trend, as indicated by the reduced diffusivity within the SC.

As well known in the literature, the exact temporal shape of the patient EGM cannot be precisely reproduced, as the details of its waveform are strongly influenced by the measurement protocol (i.e., distance and orientation of the electrode) and the local microstructure of the myocardium. Nevertheless, $\alpha =0.65$ (red trace) gives a good agreement in terms of amplitudes and importantly, it provides a correct timing of the maximum deflection point (depicted by the horizontal and vertical dashed lines in Fig. 3A), which indicates the instant of the local electrical activation of the tissue.

Setting $\alpha =0.65$ is further confirmed by the inspection of the endocardial map of the in silico LAT, shown in Fig. 3B: The activation pattern is in good agreement with clinical LATs in the peri-scar region (see Fig. 1C), with local activation values ranging from $\text {LAT}(\textbf{x})\approx 70\,\text {ms}$ in WM with a progressive activation delay within the infarcted region related to $\text {LAT}(\textbf{x})>125\,\text {ms}$, as in the in vivo recordings.

Figure  3C reports the corresponding heterogeneous diffusivity field based on Eq. (3): In regions of WM, the diffusivity map retrieves the baseline value $\beta$, lowering to a minimum of $D(\textbf{x})\approx \beta /3$ approaching the SC.

The overall spatiotemporal effect of the impairing function $F(\textbf{x})$ on action potential propagation is further illustrated in Fig. 3D, where local CV magnitude is overlaid on the streamlines of the wavefront propagation. In healthy myocardium, wavefronts propagate at approximately $0.6~\text {m/s}$, consistently with experimental measurements in post-infarction myocardium (Aronis et al. 2020). As the activation wavefront enters the SC, CV slows down progressively, reaching a minimum of roughly $0.1~\text {m/s}$. This spatial heterogeneity yields the wavefront to propagate along the interface with WM and bypass the SC, thus favoring earlier activation in adjacent viable tissue. Consequently, the SC is primarily activated retrogradely through surrounding WM regions after depolarization of the viable myocardium. Interestingly, the calibrated electrophysiology model does not include regions of complete conduction block.

Figure 3E illustrates the corresponding APD across the ventricular domain. In WM, the model reproduces the baseline dynamics with APD $\approx 190\,\text {ms}$. Still, as the wavefront approaches the scar core, a progressive increase in APD is observed, owing to the modified cell model (see Sect. 2.3.1). The mean APD rises to $270\,\text {ms}$ within the SC corresponding to an increase of roughly 40%.

## Results

### Pre-intervention: tachycardia electrophysiology pattern

**Fig. 4:**
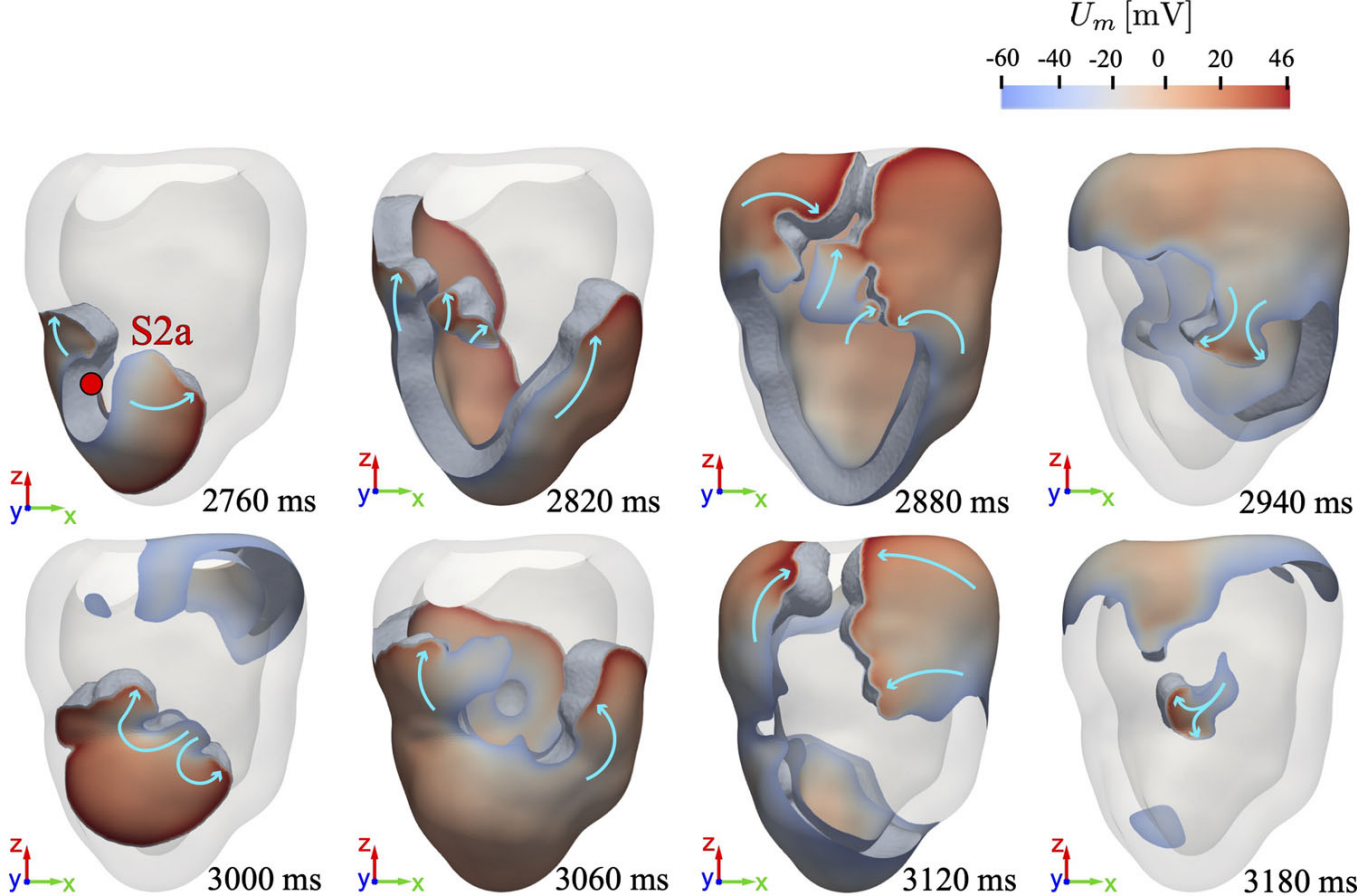
Time evolution of the induced reentry following the application of the S2a stimulus. The light blue arrows indicate the direction of wavefront propagation

Reentrant electrical activity is triggered using an S1-S2 stimulation protocol (Trayanova et al. 2024), with a pacing period of $600\,\text {ms}$ (corresponding to 100 bpm). With the aim of triggering a reentrant pattern, an ectopic stimulus (S2) is added to the sinus rhythm (S1) during the fifth cardiac cycle, and the resulting electrophysiology dynamics is tracked over the subsequent eight cycles (lasting 4.8 s). The evolution of the induced reentry is shown in Fig. 4, where the location of the ectopic beat is indicated by a red dot: Since other locations for the ectopic beat will be used in the following, we refer to this case as S2a.

Following the occurrence of the S2a stimulus, a new wavefront overlaps with the refractory tail of the fifth S1 wave, initiating a base-apex propagation. This leads to the emergence of a pair of counter-rotating scroll waves, defined following the approach proposed in Fenton and Karma (1998), which twist and propagate through the interface with WM, accompanied by partial wavefront fragmentation in the isthmus. Partial repolarization at the site of ectopic activity allows the scroll waves to reencounter and merge in the upper portion of the scar, generating a wavefront that reenters toward the right side of the isthmus, with a larger portion of the scar regaining excitability and allowing a reentrant wave to exit the isthmus. After about 3 s, approximately half of the ventricle is activated by the reentrant wavefront, while the region near the isthmus is nearly fully repolarized with scroll waves circumventing the scar.

The recurrent activation sequence following the ectopic beat constitutes a self-sustained arrhythmia within the ventricle and represents a potentially life-threatening condition for the patient. These findings confirm the clinical decision for performing RFA procedures to eliminate the arrhythmogenic substrate, given the significant risks associated with such reentrant pathways.

### Post-intervention: effect of ablation radius

**Fig. 5:**
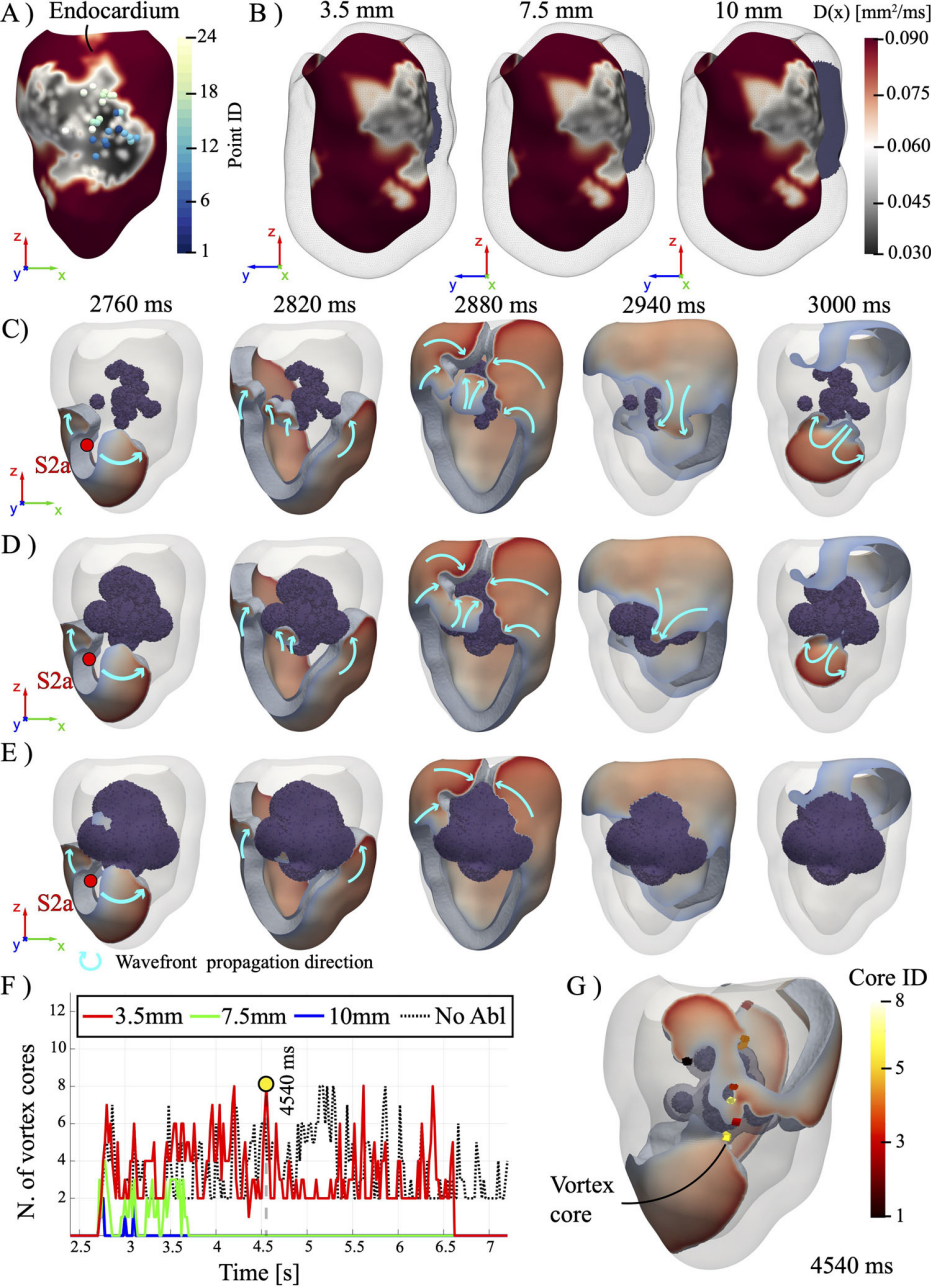
**A** Positions of the clinical points (with their relative ID). **B** Details of the cross section of the ablated volume for different values of local ablation radius, namely $R=3.5, 7.5,10\,\text {mm}$. Time evolution of the post-intervention reentry induced through S1-S2 procedure (ectopic beat S2a) for different values of the local ablation radius: **C** $R=3.5\,\text {mm}$, **D** $R=7.5\,\text {mm}$, **E** $R=10\,\text {mm}$. **F** Time evolution of the number of vortex cores with respect to the pre-intervention case (black dashed line). The yellow circle indicates the time instant of the snapshot reported in **G** for $R=3.5\,\text {mm}$

We now investigate in silico the effects of the RFA procedure performed by clinicians on the patient. From the CARTO dataset, a total of 24 ablation points are extracted and transferred onto the in silico model to retrospectively assess the effectiveness of the intervention in suppressing tachycardia activity in the patient. The clinically ablated area covers approximately $70\%$ of the identified low-voltage region, reflecting the spatial overlap between the targeted sites and the underlying substrate rather than a predefined coverage criterion, while encompassing the critical isthmus sustaining the reentrant circuit. In Fig. 5A, the recorded location of the ablating electrode is reported over the patient endocardium, together with their ID point. In the simulation, each lesion is modeled as a spherical, non-conductive region centered on the coordinates derived from clinical data.

Since the CARTO dataset does not contain data regarding the size of the lesions, the local ablation radius *R* is treated as a free parameter, and a sensitivity analysis is performed to assess the effect of ablation depth on VT suppression. The minimum radius $R=3.5\,\text {mm}$ corresponds to the catheter tip diameter employed in the clinical procedure, while the maximum radius, $R=10\,\text {mm}$, is set equal to the largest LWT. An intermediate radius of $R=7.5\,\text {mm}$ is also explored, as shown in Fig. 5B reporting the corresponding ablated volume.

For $R=3.5\,\text {mm}$, ablation remains mainly endocardial with a large portion of preserved tissue on the epicardial side, whereas for $R=7.5\,\text {mm}$, this residual conductive region is clearly less pronounced. For $R=10\,\text {mm}$, the ablated core crosses the entire myocardial wall, forming a fully transmural non-conductive region embedded within the scar. This variation in lesion geometry is also reflected in the total ablated myocardial volume, corresponding to 0.91%, 4.67%, and 8.54% of the myocardial domain for increasing values of *R*.

To test whether RFA procedure could effectively suppress arrhythmic propagation, we apply again the same S1–S2 stimulation protocol of Sect. 3.1 (with S2=S2a) to all three post-intervention models, and the corresponding short-term electrical activity is reported in Fig. 5C–E.

For the three cases, two counter-rotating scroll waves emerge, consistently with the pre-intervention simulation. For $R=3.5\,\text {mm}$ and $R=7.5\,\text {mm}$ residual conductive tissue between the ablated volume and the epicardium allows partial wavefront propagation toward the isthmus, with no major macroscopic differences between the two cases. In contrast, for $R=10\,\text {mm}$ the scroll waves shifts around the ablated region toward the atrioventricular septum. After $\approx 120~\text {ms}$ from the ectopic beat, in the $R=3.5\,\text {mm}$ and $R=7.5\,\text {mm}$ cases, the scroll waves begin to penetrate the scar region from the right side, initiating reentry on the epicardial zone of the ablated region. On the other hand, for $R=10\,\text {mm}$ the scroll-waves merge together and the repolarization phase is initiated without a reentrant circuit within the scar. Subsequently, a reentrant circuit is still present in both the $R = 3.5\,\text {mm}$ and $R = 7.5\,\text {mm}$ cases, with a slight propagation delay in the former. In contrast, the $R = 10\,\text {mm}$ case exhibits full repolarization of the ventricle, indicating successful suppression of the reentrant circuit via transmural ablation.

These findings confirm that complete transmural ablation is critical to eliminate reentrant circuits in the presence of deep scar substrates. Indeed, although sub-transmural lesions may partially modify conduction patterns, they leave viable pathways that can sustain reentrant activity lasting $\approx 4\,\text {s}$ and $\approx 1\,\text {s}$ for $R=3.5\,\text {mm}$ and $R=7.5\,\text {mm}$, respectively.

The long-term electrical activity is shown in Fig. 5F, where the temporal evolution of the number of vortex cores (i.e., scroll waves) for each ablation scenario is reported for time $\in [2400\,\text {ms},\,7200\,\text {ms}]$. The data reveal a clear dependence on the lesion radius, with suppression efficacy improving as *R* increases. Similarly to the pre-intervention case (the black dashed line corresponds to the case without ablation detailed in 3.1), for the smallest lesion size ($R = 3.5\,\text {mm}$), the number of scroll waves increases from 2 to a maximum of $\approx 8$. Importantly, a reentrant activity is observed in a time span of $\approx 4\,\text {s}$ from the occurring of the ectopic beat S2a. In the intermediate case ($R = 7.5\,\text {mm}$), the number of vortex cores initially rises from 2 to 3, but no scroll waves are observed beyond $t = 3700\,\text {ms}$. The ablation thus appears to compromise the ability of the tissue to maintain reentrant pacing at a stable frequency, ultimately leading to the spontaneous termination of VT and the reestablishment of sinus rhythm. For the largest lesion ($R = 10\,\text {mm}$), the ablation is fully effective: No scroll waves are detected throughout the simulation, demonstrating complete prevention of reentrant activity. The ablation is fully transmural and extensive enough to completely eliminate the substrate necessary for reentry.

#### Robustness of the RFA procedure

**Fig. 6:**
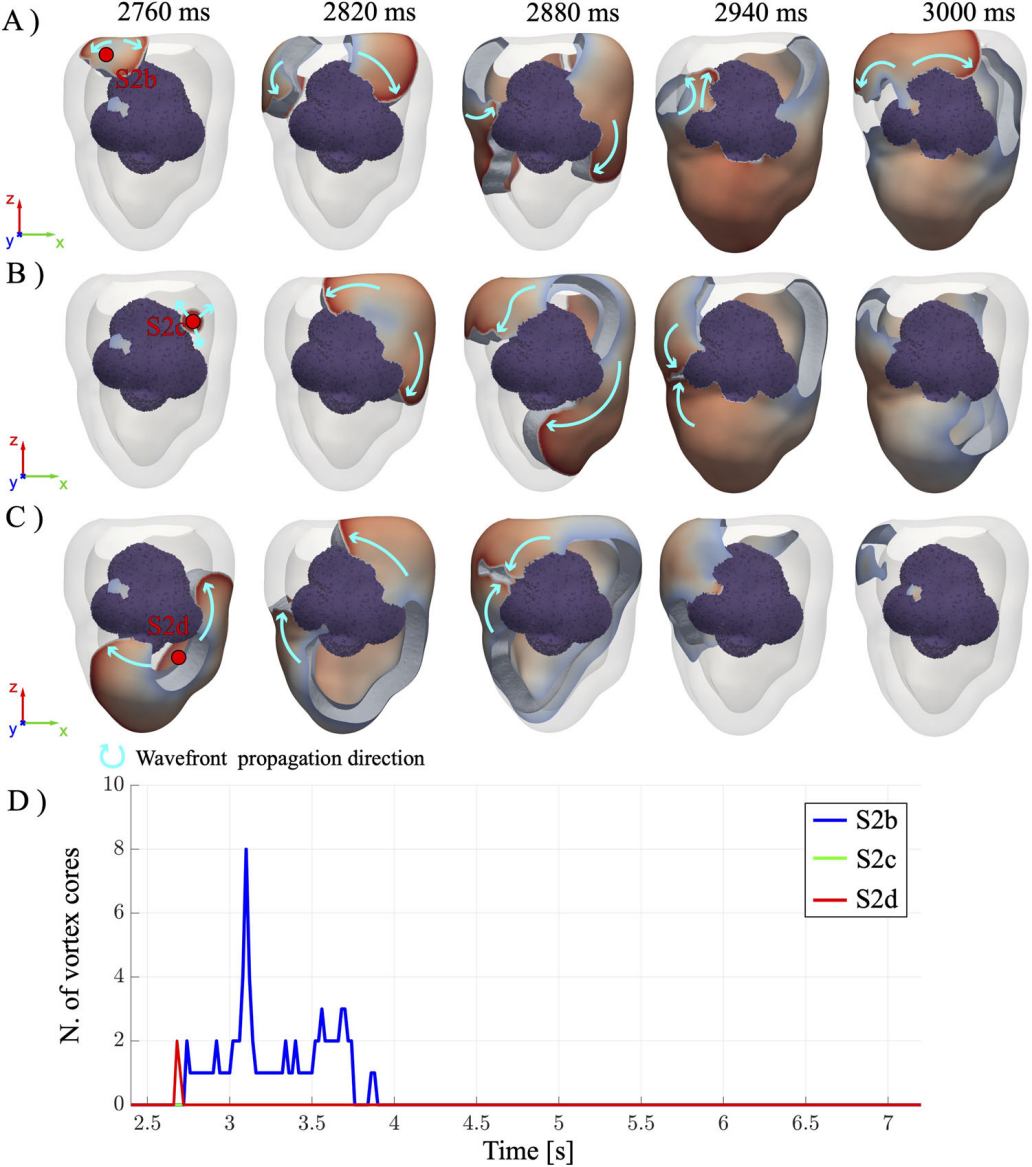
Time evolution of the post-intervention reentry induced through S1-S2 procedure for different positions of the ectopic beat: **A** S2b, **B** S2c and **C** S2d. The corresponding number of vortex cores in time is reported in **D**

In order to assess the robustness of RFA in preventing arrhythmias, we now repeat the same S1-S2 stimulation protocol but varying the location of the S2 ectopic beat around the scar. In addition to S2a, three more locations for the ectopic beat are considered (namely S2b, S2c and S2d, see Appendix C) and the corresponding activation patterns are shown in Fig. 6A, B and C for $R=10\,\text {mm}$.

**Fig. 7:**
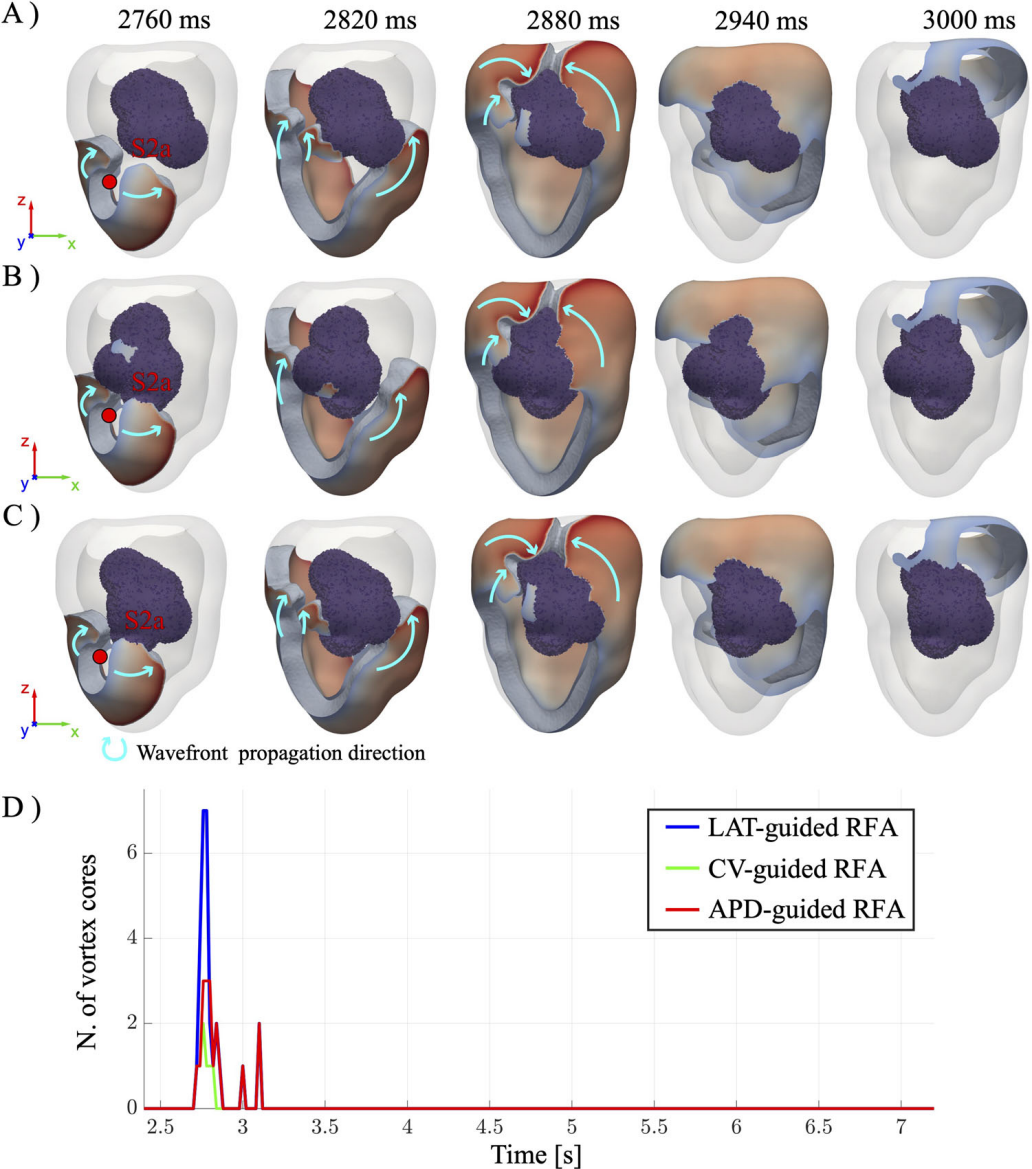
Time evolution of the post-intervention reentry induced through S1-S2 (S2=S2a) procedure for **A** LAT- **B** CV- and **C** APD-guided ablation procedures. **D** Time evolution of the corresponding number of vortex cores

Although all four ectopic stimulations ultimately lead to VT termination, S2b induces transient reentrant activity, whereas for both S2c and S2d, reentry is successfully prevented throughout the simulation.

In particular, S2b exhibits wavefront splitting upon encountering the ablation zone, thus giving rise to two counter-rotating scroll waves. One of them re-enters the scar from the top-left, marking the onset of reentry, and additional scroll waves emerge near the top-left of the scar and reactivate the adjacent working myocardium near the atrioventricular septum. After $\approx 240~\text {ms}$ from the ectopic beat, a pair of counter-rotating scroll waves is still sustained. On the other hand, although scroll wave formation also occurs for S2c and S2d, they do not form a closed-loop circuit, and they merge as the myocardium starts to repolarize. Hence, a complete suppression of reentry is observed after $\approx 240~\text {ms}$ from the ectopic beat.

These findings underscore that, while the ablation strategy procedure applied to the patient is effective, its capability in preventing the first reentry can depend on the location of the ectopic activity. Nevertheless, in the long term, VT is suppressed, as indicated by the vortex cores counting reported in Fig. 6D. In the S2b case, the onset of reentry is visible at $t \approx 2800\,\text {ms}$, marked by a rapid increase in the number of vortex cores. This activity originates from the formation of a reentrant circuit in the top-left region of the scar. However, the number of cores gradually decreases, indicating progressive wavefront disruption and eventual reentry termination. This dynamics suggests that although S2b can initiate reentrant activity, the altered post-ablation substrate does not support its long-term maintenance. For the ectopic beat S2d, such transient vortex formation is very localized in time (with two vortices detected at $t \approx 2700\,\text {ms}$) and is associated with the interaction of the first S2 wavefront with the border of the ablated region. In contrast, S2c shows no evidence of a transient reentry.

### Post-intervention: optimized ablation procedure

Let us now exploit the computational model in a more predictive manner by investigating the effectiveness of other RFA procedures involving only a subset of the ablation points used by clinicians, thereby reducing the myocardial ablation volume. The subgroup of ablation points is chosen according to three different strategies: based on (1) LAT, (2) CV, or (3) APD, which are associated with a higher susceptibility to reentry formation and can guide the ablation procedure. Specifically, among the 24 ablation locations used by the clinicians (and reproduced in silico in Sect. 3.2), we now consider only those exhibiting prolonged LAT ($>125\,\text {ms}$), slowed CV ($<{0.3}\,\text {m/s}$), or prolonged APD ($>{245}\,\text {ms}$) in sinus rhythm.

Figure 7A–C shows the resulting activation patterns following an ectopic stimulation at site S2a for the LAT-, CV- and APD-guided RFA procedures (ablation radius fixed to $R=10\,\text {mm}$). Notably, the LAT-guided procedure results in a lesion shape concentrated on the right portion of the scar, while the CV-guided procedure corresponds to an ablation volume focused on the left portion. The APD-guided approach yields a lesion morphology that approximately corresponds to the union of the LAT- and CV-guided ablated volume, with a major focus on the isthmus. Lastly, it is worth to mention that compared to the full ablation case (see previous section, $R=10\,\text {mm}$), the LAT-, CV-, and APD-guided procedures lead to a reduction of ablated volume of 23.39%, 25.16%, and 22.74%, respectively.

In all three cases, an EP wave propagates from the S2a stimulation site toward the excitable tissue. The wavefront then interacts with the ablated volume, resulting in the formation of a pair of counter-rotating scroll waves that circumvent the ablated region. In the LAT- and APD-guided ablation, the dispersion of APD and CV in the non-ablated portion of the scar leads to partial wavefront fragmentation and the formation of an additional pair of scroll waves. In contrast, no fragmentation is observed in the CV-guided procedure, as the ablated volumes occupy the left portion of the scar and eliminate the substrate necessary for secondary wavefront breakup. After about ${180}~\text {ms}$ from the ectopic beat, all scroll waves merge in the top-right portion of the ablated volume, and a large portion of the ventricle starts to repolarize. Finally, the tissue is almost fully repolarized, with no major differences among the three cases.

Importantly, the reentry is successfully prevented in all three reduced ablation strategies, highlighting the potential of this targeted approach to improve the RFA procedure by minimizing lesion volume, while preserving the effectiveness of the ablation. These results are further confirmed by Fig. 7D, where the number of vortex cores for the three RFA procedures is plotted over time. Even if some spikes (due to the interaction between the wavefront initiated by the S2a stimulus and the ablated region) are observed around $t = 2700\,\text {ms}$ for LAT- and APD-guided RFA, they are rapidly damped within approximately $400 \text {ms}$.

Lastly, the robustness of the proposed RFA procedures is tested by varying the position of the ectopic beat over the positions S2b, S2c, and S2d. As reported in the Appendix D (see Fig. 10, 11 and 12) in the long term, VT is always suppressed, i.e., for all locations of the ectopic activity and for all LAT-, CV-, and APD-guided procedures.

## Conclusion

In this study, we introduced a fast, comprehensive, data-driven in silico framework to analyze the electrophysiological behavior of infarcted myocardium in the LV and retrospectively evaluate the efficacy of catheter ablation therapy in suppressing VT. By integrating clinical electroanatomical mapping data into a calibrated monodomain model, we constructed a geometrically and electrophysiologically realistic representation of the LV, capable of capturing critical aspects of post-infarction arrhythmogenesis.

The computational model is informed by clinically measured LATs and EGMs, which are used to embed the scar morphology within the structural and electrical remodeling of the myocardium. The spatial heterogeneity in electrophysiological properties, including prolonged APD and slowed CV, has been obtained through a local modification of the tissue diffusivity and cellular ionic currents in the scar region. This approach enabled a mechanistic exploration of VT pathways that would have been challenging to obtain using clinical observation alone.

Using a standardized S1–S2 stimulation protocol mimicking clinical pacing strategies, we demonstrated that the patient-specific computational model supports the emergence and maintenance of reentrant scroll waves within the infarcted region. The induced reentrant dynamics closely resemble clinically observed VT morphologies and highlight the functional relevance of the tissue surrounding the scar as a substrate for sustained arrhythmia.

Then, virtual ablation procedures have been simulated by introducing non-conductive spherical regions centered on ablation sites adopted by clinicians for the patient under study. This approach enables a systematic evaluation of the relationship between lesion size and reentry termination. Full-thickness lesions successfully disrupt reentrant circuits and restore normal conduction, while smaller lesions fail to completely prevent reentry due to the persistence of residual conductive channels within the scar. These observations are further quantified through the analysis of vortex core dynamics, providing a metric to track the evolution and sustainability of arrhythmias.

The robustness of the ablation procedure is tested by considering different locations of the ectopic beat. While full transmural ablation generally prevents sustained reentry, certain ectopic locations (e.g., S2b) are still capable of initiating transient reentrant activity that eventually lead to VT termination in the long term. This underscores the importance of lesion placement strategies that consider both anatomical scar distribution and the functional characteristics of the surrounding myocardium.

Lastly, the computational model has been exploited to investigate optimized ablation strategies, where only a subset of the ablation points is considered. These physiologically informed strategies achieve effective reentry suppression while reducing total ablation volume by up to $\approx 25\%$ and demonstrate the feasibility of model-informed optimization of lesion placement, potentially enhancing procedural efficiency and preserving healthy myocardium.

It is worth noting that the combination of the proposed data-driven procedure with a GPU-accelerated electrophysiology solver enables rapid calibration and efficient utilization of the patient-specific computational model. In particular, the effectiveness of a given ablation strategy can be assessed within just a few minutes, and the simultaneous use of multiple GPU devices allows for the parallel evaluation of alternative ablation plans. This numerical approach not only accelerates the overall time-to-solution but also enables in silico testing of the ablation within short clinical timeframes. The proposed methodology, therefore, represents a significant step toward in silico–assisted clinical decisions, with the potential to improve patient long-term outcomes identifying critical isthmus and reducing high VT ablation recurrences.

***Limitations and Perspectives***

Despite its promising results, it should be considered that the present work deliberately considers the ventricular geometry based exclusively on electroanatomical mapping data, which is limited to the endocardial surface. Consequently, mid-myocardial and epicardial structures—potentially critical to arrhythmogenesis—are not directly captured, but wall thickness is inferred through rule-based extrapolations. While this approach allows for fast and automated model generation, it cannot account for individual anatomical features that may influence electrical conduction (Trayanova et al. 2024). While this simplification may limit anatomical fidelity in regions of epicardial remodeling, the geometric pipeline is fully automated, clearly described, and readily reproducible across datasets, making it suitable for integration into clinical modeling workflows for precision medicine (Peirlinck et al. 2021).

In addition, the use of a scalar viability field and an isotropic monodomain formulation enables computational efficiency and interpretability, but comes at the cost of reduced physiological detail. Microstructural features such as fiber orientation, conduction anisotropy, and heterogeneous interstitial fibrosis (Bansmann et al. 2024)—now restricted only to the isthmus zone—should be included to augment the model reliability. Moreover, while the conductivity profile is modulated through an empirically calibrated parameter, a more rigorous approach would involve the use of inverse problem techniques to infer spatially resolved conductivity distributions (Barone et al. 2020; Wallman et al. 2014). Incorporating such methods would enhance the mechanistic interpretability of the model and improve parameter identifiability.

Moreover, ablation lesions are idealized as spherical, fully non-conductive volumes with variable transmurality. While this approach enables systematic exploration of lesion effectiveness, it does not replicate the variability in lesion geometry and shape observed in vivo, which is influenced by factors such as catheter contact, tissue thickness, and thermoelastic dynamics (Bianconi et al. 2025; Molinari et al. 2022a, b). In addition, the reconstruction of the clinical ablation is based on the recorded catheter positions, which define the targeted regions without fully specifying the resulting lesion geometry or accounting for potential gaps and heterogeneous tissue response. As a consequence, the in silico representation should be interpreted as a controlled framework to evaluate the functional role of the clinically selected targets, rather than a detailed reproduction of lesion formation in vivo.

Finally, future developments should aim to more accurately reproduce the fine-scale features of the recorded VT dynamics, including the VT cycle length observed in the patient, which is currently underestimated in the present analysis. Achieving this objective will likely require a more refined electrophysiological parameterization, together with a more detailed incorporation of patient-specific fibrosis and myocardial microstructural heterogeneity (Trayanova et al. 2017; Sung et al. 2021). In particular, the absence of high-resolution data on fibrosis density, tissue micro-architecture, and patient-specific fiber orientation (e.g., due to the lack of DT-MRI or CT imaging) represents a key limitation. Consequently, the present model employs a functional representation of the scar as a heterogeneous, arrhythmogenic substrate capable of sustaining reentry, rather than reconstructing a fully anatomically detailed substrate with explicitly resolved structural discontinuities. These simplifications may influence conduction velocity, wavefront dynamics, and reentry stability, thereby contributing to the observed discrepancy in VT cycle length and limiting the quantitative predictive capability of the model. Addressing these limitations is the focus of ongoing work by the authors, aimed at improving model fidelity and patient-specific characterization.

**Fig. 8:**
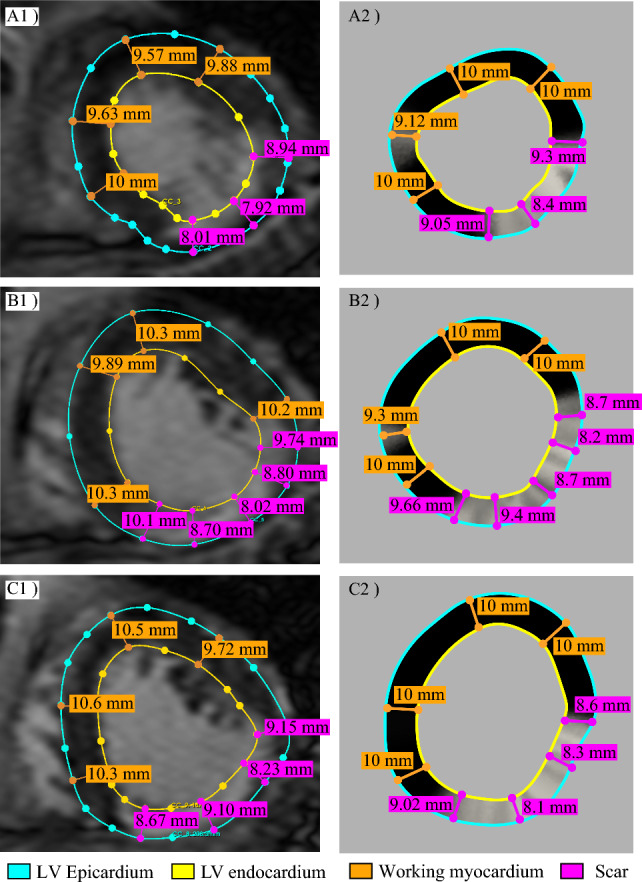
Comparision between MRI slices acquired in vivo vs geometry slices obtained in silico for (**A**1, **A**2) distal, (**B**1, **B**2) medial and (**C**1, **C**2) proximal portion of the ventricle in the long-axis direction. Measuring lines are reported in magenta for the scar region and in orange for the working myocardium, respectively

**Fig. 9:**
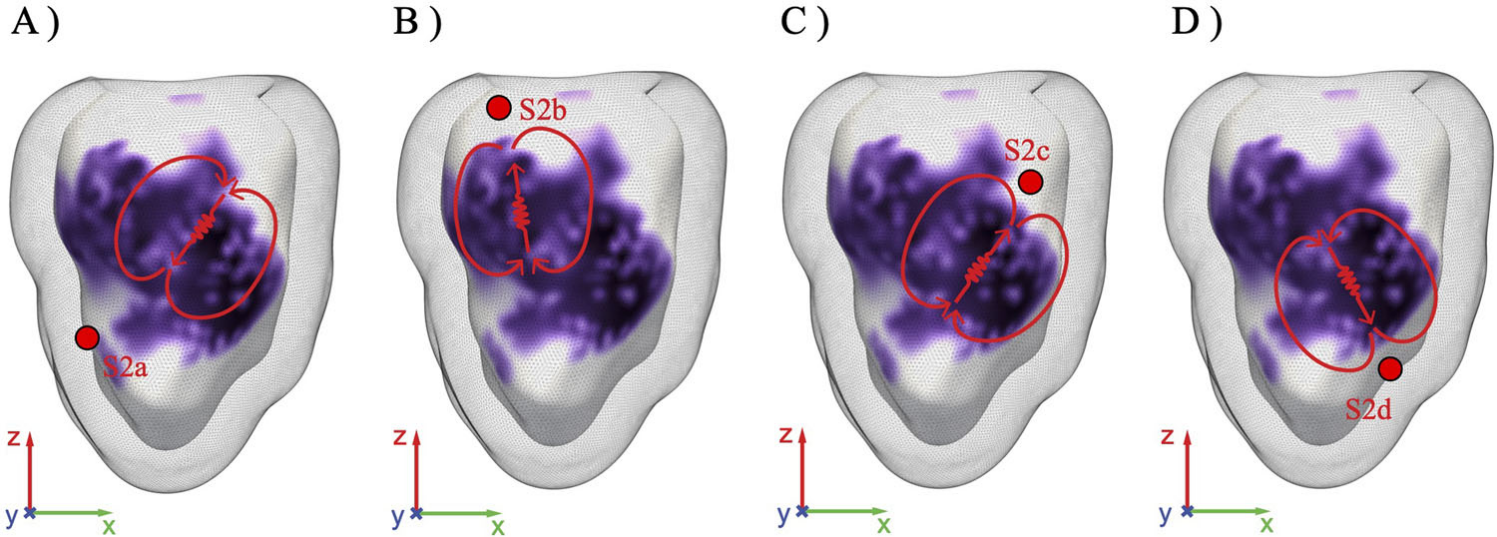
Locations of the ectopic S2 stimulus overlaid on the endocardial surface: **A** S2a with $\tau _{\text {S2}}=285\,\text {ms}$, **B** S2b with $\tau _{\text {S2}}=325\,\text {ms}$, **C** S2c with $\tau _{\text {S2}}=350\,\text {ms}$ and **D** S2d with $\tau _{\text {S2}}=270\,\text {ms}$. The scar region is indicated by the purple region, and the red arrows indicate the reentry circuits resulting from each S1–S2 stimulation protocol

## Figures and Tables

**Fig. 10:**
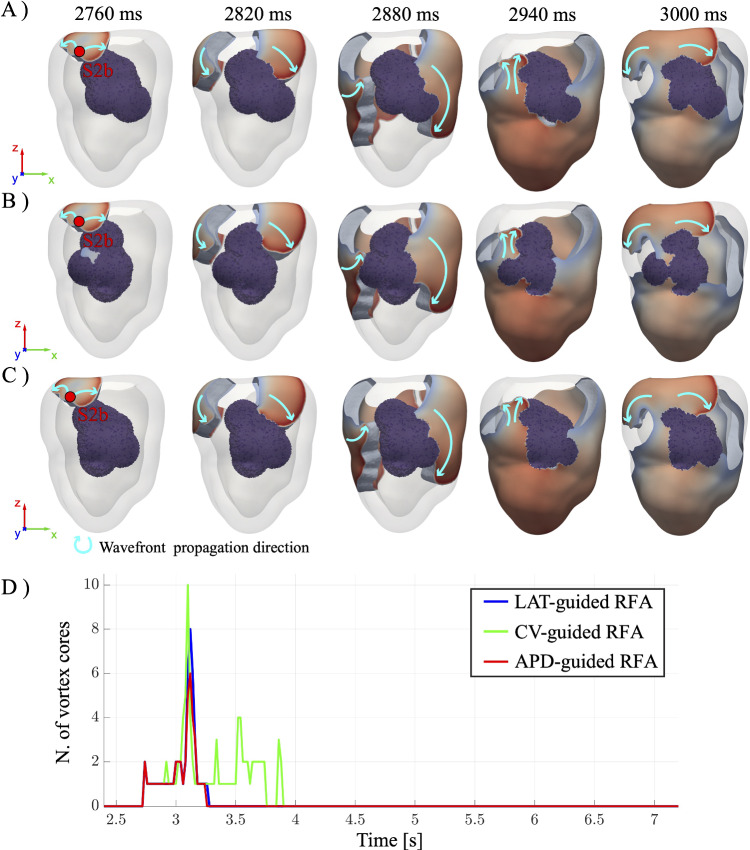
Same as Fig. 7, but for S2=S2b

**Fig. 11:**
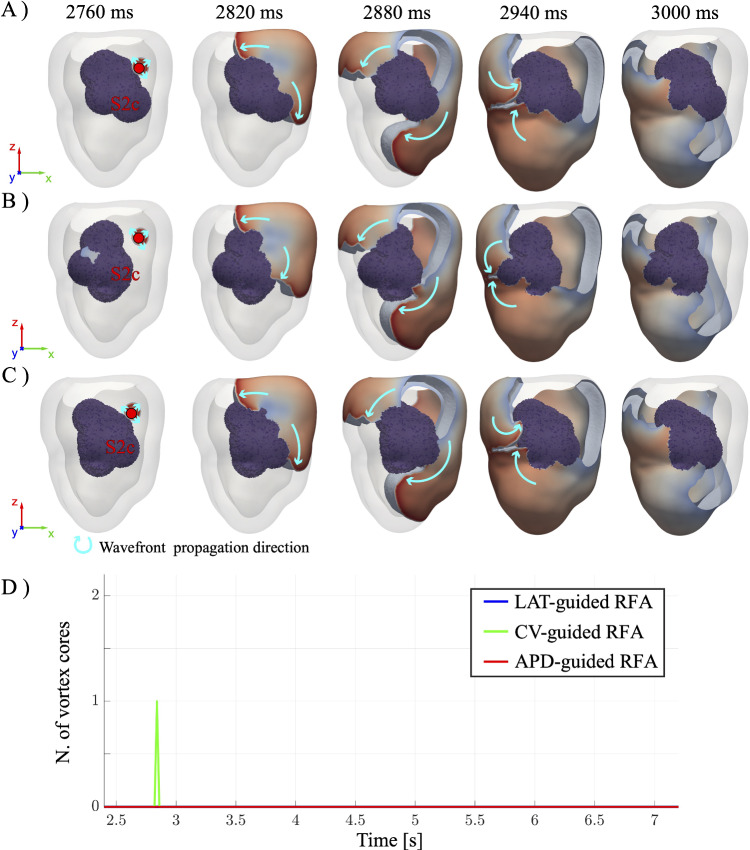
Same as Fig. 7, but for S2=S2c. The empty final panel indicates the absence of vortices, with the counter remaining at zero

**Fig. 12:**
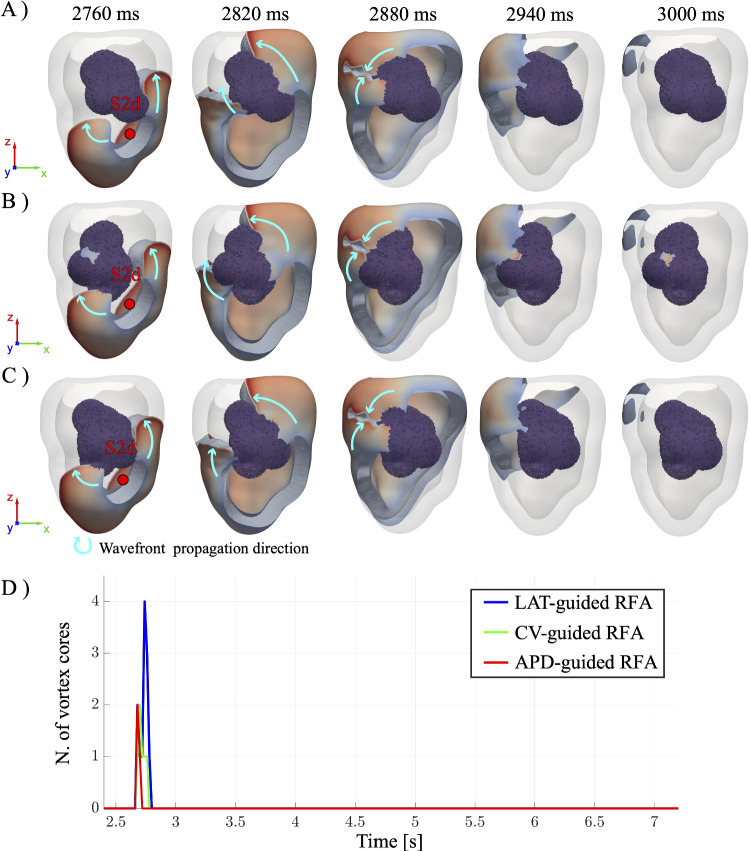
Same as Fig. 7, but for S2=S2d

## Data Availability

All relevant data supporting the findings of this study will be made available upon reasonable request.

## Appendix A: Validation of altered myocardium thickness

Figure 8 shows available cardiac MRI, alongside the corresponding cross sections of the reconstructed computational geometry obtained as detailed in Sect. 2.2.1. It should be remarked that this MRI dataset is characterized by a limited through-plane resolution, with a slice thickness along the *z*-direction of approximately 9 mm, which does not allow for a reliable patient-specific three-dimensional reconstruction of the transmural wall profile. The MRI slices clearly display a region of preserved healthy myocardium, corresponding to the largest wall thickness (approximately 10 mm), progressively reducing toward the infarcted region down to about 8 mm. The same spatial trend is reproduced in the computational geometry generated via Eq. (1) , where the local wall thickness smoothly transitions from working myocardium to scar core according to the impairing function $F(\textbf{x})$. This comparison supports that, despite the limited *z*-resolution of the MRI and the absence of a full volumetric reconstruction, the adopted rule-based approach provides a geometrically consistent representation of the patient-specific wall thickness in the regions analyzed.

## Appendix B: Four variable minimal model for MI

We report here the details of the cellular model derived from (Fenton et al. 2013) and the modifications which have been introduced to model MI. The system of equations  (2) governing the propagation of electrophysiological waves within the infarcted ventricle is given by:

4 $$\begin{aligned} \begin{aligned} \partial _t U_m&=\nabla \cdot \left( \textbf{D}(F(\textbf{x}))\nabla U_m\right) -\left( J_{f i}(\textbf{x})+J_{s o}(\textbf{x})+J_{s i}(\textbf{x}) \right) \\ \partial _t v&= \left( 1-H\left( U_m-\theta _v\right) \right) \frac{\left( v_{\infty }-v\right) }{\tau _v^{-}}-\frac{H\left( U_m-\theta _v\right) v}{\tau _v^{+}}\\\partial _t w&=\left( 1-H\left( U_m-\theta _w\right) \right) \frac{\left( w_{\infty }-w\right) }{\tau _w^{-}}-\frac{H\left( U_m-\theta _w\right) w}{\tau _w^{+}}\\ \partial _t s&=\frac{1+\tanh \left( k_s\left( U_m-u_s\right) \right) }{2\tau _s} - \frac{s}{\tau _s},\\\end{aligned} \end{aligned}$$

where the impairing function $F(\textbf{x})$ (see Fig. 2B,C) is embedded in the ionic density currents $J_{f i}$, $J_{so}$ and $J_{si}$ as

5 $$\begin{aligned} J_{f i}(\textbf{x}) &  = \left[ -H\left( U_m-\theta _v\right) \left( U_m-\theta _v\right) \left( u_u-U_m\right) \frac{v}{\tau _{f i}} \right] R(\textbf{x}) \nonumber \\ J_{s o}(\textbf{x}) &  =\left[ \left( 1-H\left( U_m-\theta _w\right) \right) \frac{\left( U_m-u_o\right) }{\tau _o}+\frac{H\left( U_m-\theta _w\right) }{\tau _{s o}}\right] R(\textbf{x})\nonumber \\ J_{s i}(\textbf{x}) &  = \left[ -H\left( U_m-\theta _w\right) \frac{w s}{\tau _{s i}} \right] R(\textbf{x}) \end{aligned}$$

where $R(\textbf{x}) = 1-\alpha (1-F(\textbf{x}))$, with the scaling parameter $\alpha$ set as reported in Sect. 2.3.2. The voltage-dependent time constants are set as in Fenton et al. (2013)

6 $$\begin{aligned} \tau _v^{-}(U_m) &  =\left( 1-H\left( U_m-\theta _v^{-}\right) \right) \tau _{v 1}^{-}+H\left( U_m-\theta _v^{-}\right) \tau _{v 2}^{-}\nonumber \\ \tau _w^{+}(U_m) &  =\tau _{w 1}^{+}+\left( \tau _{w 2}^{+}-\tau _{w 1}^{+}\right) \frac{\tanh \left( k_w^{+}\left( U_m-u_w^{+}\right) \right) +1}{2} \nonumber \\ \tau _w^{-}(U_m) &  =\tau _{w 1}^{-}+\left( \tau _{w 2}^{-}-\tau _{w 1}^{-}\right) \frac{\tanh \left( k_w^{-}\left( U_m-u_w^{-}\right) \right) +1}{2} \nonumber \\ \tau _{s o}(U_m) &  =\tau _{s o 1}+\left( \tau _{s o 2}-\tau _{s o 1}\right) \frac{\tanh \left( k_{s o}\left( U_m-u_{s o}\right) \right) +1}{2} \nonumber \\ \tau _s(U_m) &  =\left( 1-H\left( U_m-\theta _w\right) \right) \tau _{s 1}+H\left( U_m-\theta _w\right) \tau _{s 2} \nonumber \\ \tau _o(U_m) &  =\left( 1-H\left( U_m-\theta _o\right) \right) \tau _{o 1}+H\left( U_m-\theta _o\right) \tau _{o 2}.\nonumber \\ \end{aligned}$$

with the asymptotic values of $v_\infty$ and $w_\infty$ equal to

7 $$\begin{aligned} \begin{aligned} v_{\infty }&= {\left\{ \begin{array}{ll}1, & U_m<\theta _v^{-} \\ 0, & U_m \ge \theta _v^{-}\end{array}\right. } \\ w_{\infty }&=\left( 1-H\left( U_m-\theta _o\right) \right) \left( 1-\frac{U_m}{\tau _{w \infty }}\right) +H\left( U_m-\theta _o\right) w_{\infty }^* \end{aligned} \end{aligned}$$

## Appendix C : Sensitivity on the location of the ectopic activity in the S1-S2 stimulation protocol

To investigate the robustness of the ablation procedure in the suppression of the VT induced by a S1-S2 stimulation protocol, we consider four alternative S2 stimulation sites—namely S2a, S2b, S2c, and S2d—whose locations are illustrated in Fig. 9. Each panel depicts the spatial relationship between the ectopic beat and the scar (highlighted in purple), alongside a sketch of the resulting reentrant circuits, indicated by red arrows. To account for the altered propagation dynamics associated to each ectopic beat, the timing of each stimulus is adjusted relative to the baseline case (see Fig. 9) and the corresponding delays $\tau _{\text {S2}}$ are reported in the figure caption.

## Appendix D: Robustness of the LAT-, CV-, and APD-guided RFA procedures

The robustness of the proposed LAT-, CV-, and APD-guided ablation procedures is investigated by varying the location of the ectopic beat (as done for the baseline RFA in Sect. 3.2). In addition to the case S1-S2a already reported in Sect. 3.3, the electrical activity of the three procedures is shown in Fig. 10 (for S2b), Fig. 11 (for S2c) and Fig. 12 (for S2d).

## Abbreviations

APD: Action potential duration
LWT: Local wall thickness
CV: Conduction velocity
MI: Myocardial Infraction
EAM: Electroanatomical mapping
RFA: Radiofrequency ablation
EGM: Electrogram
SC: Scar core
LV: Left ventricle
VT: Ventricular tachycardia
LAT: Local activation time
WM: Working myocardium
